# Patient-reported outcomes in Gaucher’s disease: a systematic review

**DOI:** 10.1186/s13023-023-02844-w

**Published:** 2023-08-25

**Authors:** Junchao Feng, Zhongchun Gao, Zhao Shi, Yue Wang, Shunping Li

**Affiliations:** 1 Centre for Health Management and Policy Research, School of Public Health, Cheeloo College of Medicine, Shandong University, Jinan, 250012 China; 2 NHC Key Lab of Health Economics and Policy Research (Shandong University), Jinan, 250012 China; 3 Center for Health Preference Research, Shandong University, Jinan, 250012 China; 4 Qingdao Hospital, University of Health and Rehabilitation Sciences (Qingdao Municipal Hospital), Qingdao, 266011 China

**Keywords:** Quality of life, Patient-reported outcomes, Literature review, Rare disease, Gaucher’s disease

## Abstract

**Background:**

Gaucher’s disease (GD), a rare condition, represents the most common lysosomal storage disorder. The cardinal manifestations of GD are fatigue, hepatosplenomegaly, anemia, thrombocytopenia, bone pain, and bone infarction, thereby culminating in a marked deterioration of patients’ quality of life (QoL). Patient-reported outcomes (PROs) offer valuable insights into the impact of GD on patients’ QoL and symptoms. This systematic review aimed to identify and analyze PROs and outcome measures in GD patients.

**Methods:**

We systematically searched PubMed, Web of Science Core Collections, EMBASE, SCOPUS, Cochrane Library, PsycINFO, Wan Fang Data, China National Knowledge Infrastructure (CNKI), and the Chinese Biomedical Literature Database (CBM). The methodological quality of the included studies was assessed using a mixed methods assessment tool.

**Results:**

A total of 33 studies were identified, encompassing 24 distinct patient-reported outcome instruments, with the most frequently employed instrument being the SF-36. The study designs included eighteen cross-sectional studies, seven pre- and post-intervention investigations, three randomized controlled trials, two cohort studies, two qualitative inquiries, and one validation study. These studies explored diverse domains such as the QoL and cardinal symptoms (e.g., fatigue, pain, bleeding, cognition, social relationships, and psychological functioning) in patients with GD. Furthermore, significant attention was directed towards the appraisal of the therapeutic benefits of various interventions in patients with GD. A novel GD-specific instrument has also been developed, which has two applied versions: a 24-item variant for routine clinical monitoring and a 17-item form for use in clinical trials.

**Conclusion:**

PROs have garnered increased attention and concern in the realm of GD. Despite this progress, it is noteworthy that the instruments used to measure PROs in GD are still predominantly generic instruments. While researchers have endeavored to develop and validate a disease-specific instrument, currently the use of this instrument is limited. Owing to several challenges, including the small number of patients, heterogeneity of the disease, and cross-regional discrepancies in study findings, GD poses substantial difficulties in the measurement of QoL and development of instruments. Consequently, patients with GD require more dependable measurement instruments that accurately reflect their QoL, efficacy of treatment, and facilitate healthcare decision-making.

**Supplementary Information:**

The online version contains supplementary material available at 10.1186/s13023-023-02844-w.

## Background

Gaucher disease (GD) is a rare autosomal recessive genetic disorder caused by a pathogenic variation in the *GBA1* gene [1]. GD is the most common lysosomal storage disorder with an estimated incidence of around 1 in 40,000–60,000 individuals in the general population [2, 3]. The GD phenotype is heterogeneous and clinically divided into three subtypes (1, 2, and 3), with GD-1 accounting for the majority of cases at approximately 90–95% [4]. GD-1, also known as non-neuropathic GD, is distinct from types 2 and 3, referred to as neuropathic GD [5]. The most common symptoms of GD include fatigue, hepatosplenomegaly, anemia, reduced platelet count (leading to easy bruising and prolonged clotting time), bone pain, and bone infarctions that often damage the shoulder or hip joints [5]. These symptoms have a profound impact on patients’ quality of life (QoL) in areas such as impairment of daily activities, self-care, body image, relationships with family, work performance, or school [6, 7]. At present, the primary clinical approaches for GD management are substrate-reduction therapy (SRT) and enzyme replacement therapy (ERT) [8]. Although both ERT and SRT are efficacious treatments, they are invasive, costly, and require patients to modify their work and personal schedules [6].

Patient-reported outcomes (PROs) have been defined as any information “of a patient’s health condition that comes directly from the patient, without interpretation of the patient’s response by a clinician or anyone else” [9]. PROs have gained increasing prominence in the health technology assessment process, following the rise of a patient-centered approach to healthcare [10]. PROs data can provide valuable evidence to facilitate shared decision making, labeling statements, clinical guidelines, and health policy [11]. Owing to the special characteristics of rare diseases, such as high unmet need, severity and debilitating nature of the condition, and a dearth of appropriate data, there is a heightened necessity to supplement traditional measurement methods with PROs in a creative and pragmatic manner [12]. The principal methods employed for gathering PROs include qualitative interviews and patient-reported outcome measures (PROMs), with the latter being the primary measurement instrument. PROMs are validated questionnaires that possess robust psychometric properties and are commonly implemented in clinical trials and disease management [9]. In summary, utilizing PROs to comprehend the health status and treatment outcomes of GD patients is a critical step towards enhancing patient care and healthcare decision-making.

Numerous studies have investigated patients with GD using PROMs or qualitative methods. Self-reported symptoms [13] of GD patients (e.g., fatigue, pain, bleeding swelling, and anemia) have a serious impact on patients’ QoL [14, 15], mental health [6, 16], social functioning [17], and cognitive abilities [18]. ​Some studies have also illustrated the effects of ERT and other interventions on patients’ QoL [19, 20]. In addition, a disease-specific instrument for GD was developed and validated [21]. However, comprehensive reviews of the use of PROs and core outcomes in GD patients have been limited. Therefore, we conducted a mixed-methods systematic review to conceptualize the stipulated overall understanding of QoL in GD and identify challenges to greater implementation and interpretation that can benefit from further research.

## Methods

This review was conducted in accordance with the PRISMA guidelines [22]. The review protocol was registered with PROSPERO (https://www.crd.york.ac.uk/prospero/; Registration ID: CRD42020192027).

### Search strategy

English and Chinese databases were included to consider the linguistic expertise of the review authors. We systematically searched the following databases: PubMed, Web of Science Core Collections, EMBASE, SCOPUS, Cochrane Library, Wan Fang Data, China National Knowledge Infrastructure, and Chinese Biomedical Literature Database. All dissertations from the inception of each database until January 2023 were considered. Two groups of search terms (Additional file 1) were used:1) PRO related terms and 2) GD-related terms. Moreover, the references were manually searched for additional papers. As our search did not include many clinical trials, it was expanded to include other study designs (e.g., pre-post studies, cross-sectional studies, and qualitative studies).

### Selection and data extraction

Studies were independently screened by two reviewers (JCF or YW), based on whether their title and abstract adhered to the selection criteria (GD, English or Chinese studies, and related to PROs or QoL) to determine their eligibility for inclusion in the full-text review. Studies were excluded for the following reasons: non-GD disease, not in English or Chinese, case study design, out-of-scope population (e.g., non-human subjects), and secondary paper. The publication years were not limited. Subsequently, the full texts of potentially eligible studies were searched and independently screened by the two reviewers. After each reviewer completed the two processes, any differences were resolved through discussion or consultation with a third investigator (SPL). The two independent reviewers then extracted the following data from each study: study design, patient characteristics, treatments, PROMs, and primary outcomes.

### Methodological quality appraisal

The methodological quality of the included studies was independently assessed by two review authors (JCF and YW) using the Mixed Methods Assessment Tool (MMAT) [23]. MMAT is a unique tool that allows the reviewers to assess the methodological quality of studies of different designs (qualitative, quantitative and mixed methods studies) in systematic mixed research reviews [24]. The MMAT guidelines discourage the calculation of an overall score from the scores for each criterion and recommend a more detailed presentation of the scores for each criterion to better understand the quality of the included studies. The exclusion of studies with low methodological quality is usually discouraged. Because there are only a few criteria per domain, a descriptor, such as a star (*) or a percentage, can be used to indicate the score.

## Results

### Results from the literature searches

**Fig. 1:**
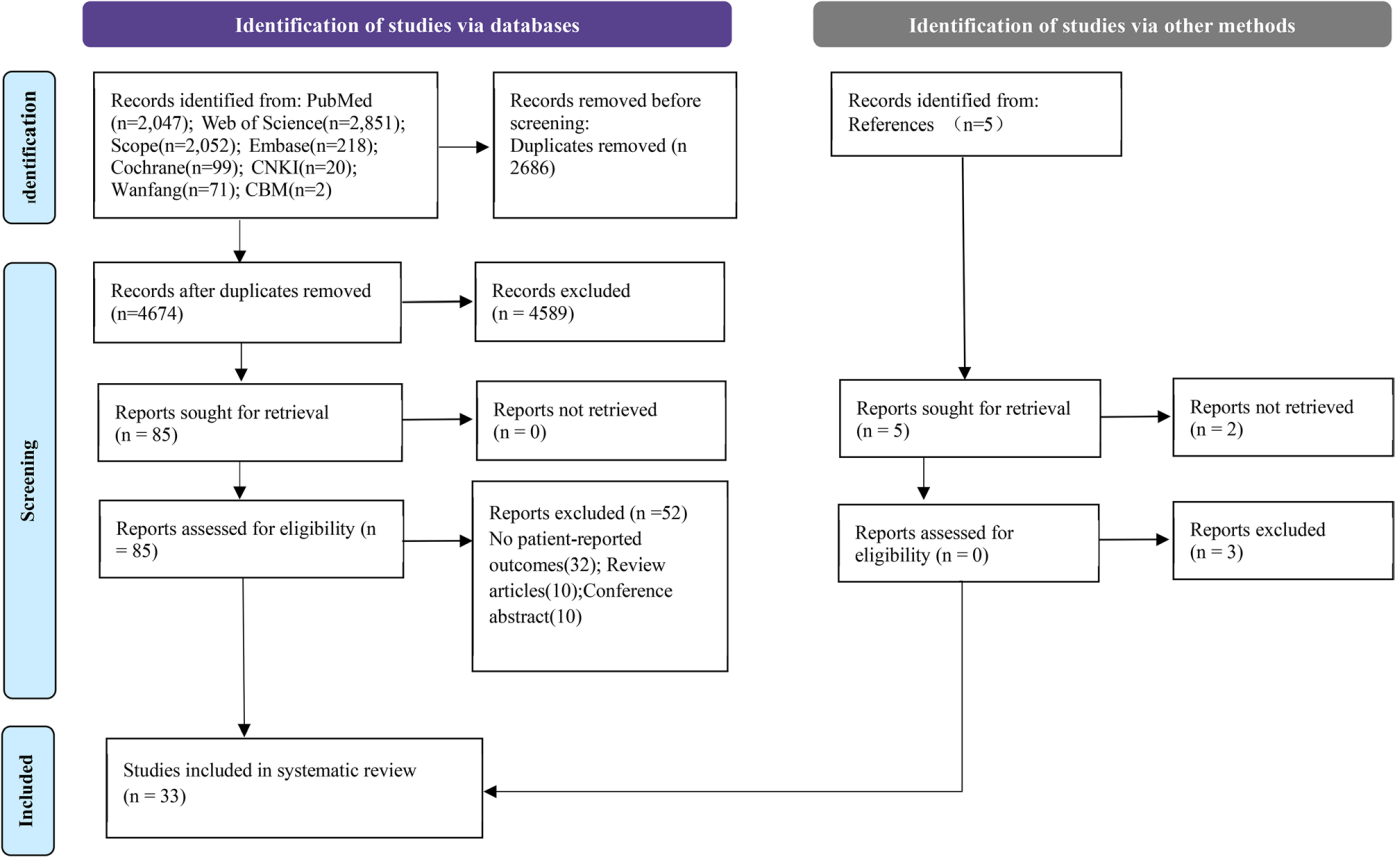
PRISMA flowchart describing the identification, selection and inclusion of studies on PRO in Gaucher’s disease

A total of 7360 studies (including 93 Chinese articles) were identified, with 4674 remaining after the removal of duplicates, and 85 remaining after the review of titles and abstracts. Of these, 85 met the full-text review criteria, and 33 met the inclusion criteria. Figure 1 summarizes the flow of the articles through the selection process.

**Fig. 2:**
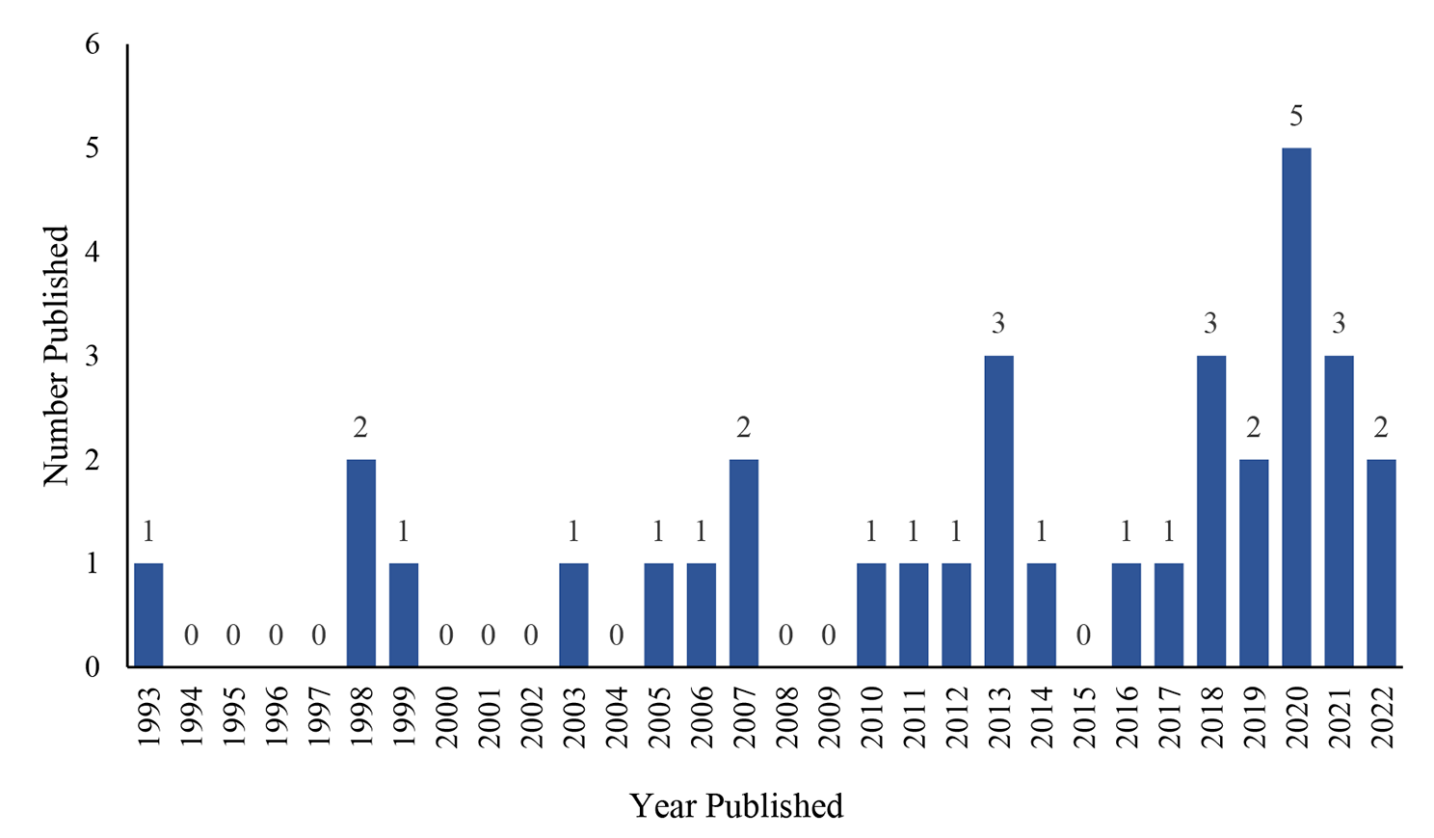
Number of GD publications per year involving PROs

As shown in Fig. 2, there was a clear trend toward an increase in the number of published studies with PROs endpoints over time, especially since 2016. The studies were conducted mainly in high-income countries, with the top three being the United States, Spain, and Israel; the number of studies in China was equal to that in Israel because of the inclusion of relevant studies in Chinese. PROMs measuring the overall QoL were used more frequently than symptom-directed instruments, and the most commonly used instrument was the Medical Outcomes Study Health Survey Short Form-36 Item (SF-36), which was employed in 17 studies.

The general characteristics of the included studies are summarized in Table 1. Of the thirty-three included studies, eighteen were cross-sectional, seven were pre- and post-intervention investigations, three were randomized controlled trials (RCT), two were cohort studies, two were qualitative studies, and one was a validation study. The following is a summary of the results of the literature review of the different biomedical literature databases. It is organized by the study design into clinical trials, longitudinal observational studies, cross-sectional studies, and pre- and post-intervention investigations.

**Table 1:** Characteristics of included studies

Author (Year) *(Reference)*	Study design	Disease types (1, 2 and 3)	Sample size	PROM	Quality assessment
Verderese C L (1993) [41]	Pre- and post-intervention	1	12	Self-made questionnaire	*** Small sample size and lack of inclusion and exclusion criteria; Measures not validated and reliability tested
Damiano A M (1998) [20]	Cross-sectional	1	212	SF-36	*****
Hayes R P (1998) [13]	Qualitative interview	1	16	-	**** Insufficient quotes provided to prove the theme
Masek B J (1999) [15]	Pre- and post-intervention	1	25	SF-36; SCL-90-R; AAC	*****
Pastores G M (2003) [39]	Cross-sectional	1	55	SF-36	*** lack of inclusion and exclusion criteria; Not reported with the survey method
Giraldo P (2005) [14]	Pre- and post-intervention	1	69	SF-36	*****
Packman W (2006) [16]	Cross-sectional	NA	28	MMPI-2	*****
Elstein D (2007) [25]	RCT	1	36	SF-36	**** Open-label clinical trial
Weinreb N J (2007) [42]	Pre- and post-intervention	1	32	SF-36	**** > 20% drop-out rate
Packman W (2010) [6]	Qualitative description	NA	28	-	**** Unclear methods for qualitative data analysis
Deegan P B (2011) [30]	Cross-sectional	1&3	100	EQ-5D; BPI	*****
Samuels N (2012) [45]	Pre- and post-intervention	NA	12	SF-12; FACIT-F;	*** Small sample size and lack of inclusion and exclusion criteria; Failure to control confounders
Deroma L (2013) [28]	Cohort	1	34	SWB	**** Measures not validated and reliability tested
Oliveira F L (2013) [32]	Cross-sectional	1&3	21	SF-36	*****
Kamusheva M (2013) [31]	Cross-sectional	NA	3	SF-36	** Small sample size and lack of inclusion and exclusion criteria; Failure to control confounders; Not reported with the survey method
Medrano-Engay B (2014) [27]	RCT	1	8	SF-36	** Not properly randomized Open-label clinical trial > 20% drop-out rate
Giraldo P (2016) [17]	Cross-sectional	1	108	SF-36; VAS for pain	*****
Devigili G (2017) [38]	Cross-sectional	1	25	NPSI	**** lack of inclusion and exclusion criteria;
Cerón-Rodríguez M (2018) [43]	Pre- and post-intervention	1	5	LPPS	*** Small sample size and lack of inclusion and exclusion criteria; Risk of bias in parental proxy responses
Remor E (2018) [7]	Cross-sectional	NA	20	PedsQL 4.0	**** Risk of bias in parental proxy responses;
Lukina E (2019) [26]	RCT	1	26	SF-36; FFS	*** Open-label clinical trial; > 20% drop-out rate
Roca-Espiau M (2019) [33]	Cross-sectional	1,2&3	25	SF-36	*****
Wilke M (2019) [40]	Cross-sectional	1	23	MoCA; ESS; BDI; UMSARS; RBD;	*****
Alioto A G (2020) [34]	Cross-sectional	1	32	PedsQL 4.0; BASC-2	*****
Cohen D (2020) [29]	Cohort	NA	48	EQ-5D; VAS for pain; HHS	*****
Dinur T (2020) [46]	Cross-sectional	1	192	GD1-PROM	*****
Tantawy, A. A. G (2020) [18]	Cross-sectional	1&3	24	BDI	*****
Li HM* (2020) [35]	Cross-sectional	1	22	SF-36	*****
De Mello R a F(2021) [36]	Cross-sectional	NA	18	SF-36	*****
Hu J (2021) [19]	Cross-sectional	NA	5	EQ-5D	*** Small sample size and lack of inclusion and exclusion criteria; Risk of bias in parental proxy responses;
Qi X (2021) [37]	Cross-sectional	NA	40	SSRS; PSQI; SF-36	*****
Elstein D (2022) [21]	Validation	1&3	124	GD-1 PROM; SF-36	*****
Lu H * (2022) [44]	Pre- and post-intervention	NA	6	SF-36	*** Small sample size and lack of inclusion and exclusion criteria; Risk of bias in parental proxy responses;

* Chinese article; NA: Not reported in study

**Abbreviations: SF-36**: 36-item Short Form Health Survey; **SCL-90-R**: Symptom Checklist-90R; **ACC**: Adult Activities Checklist; **MMPI-2**: The Minnesota Multiphasic Personality Inventory; **EQ-5D**: EuroQol Five Dimensions Questionnaire; **BPI**: Brief Pain Inventory; **SF-12**: The Short-Form Health Survey-12 items; **FACIT-F**: Functional Assessment of Chronic Illness Therapy-Fatigue measure; **SWB**: Subjective Well-Being; **VAS**: visual analogue scale ;**NPSI**: Neuropathic Pain Symptom Inventory; **LPPS**: The Lansky Play-Performance Scale; **PedsQL 4.0**: Pediatric Quality of Life Inventory; **FFS**: Fatigue Severity Scale; **MoCA**: Montreal Cognitive assessment; **ESS**: Epworth sleep Scale; **BDI**: Beck Depression Inventory; **UMSARS**: Unified Multiple System Atrophy Rating Scale ; **RBD**: REM sleep behavior disorder; **BASC-2**: Behavior Assessment System for Children; **HHS**: Harris Hip Score; **GD1-PROM**: Gaucher disease type 1-specific patient-reported outcome measures; **SSRS**: Social Support Rating Scale; **PSQI**: Pittsburgh sleep quality Index

### Methodological quality of the included studies

The overall quality score was 100% (5 of 5 criteria met) for seventeen studies (51.5%), 80% for seven (21.2%), 60% for seven (21.2%), 40% for two (6.1%), and 20% for zero (0%) studies, as shown in Table 1. The most common causes for downgrading the quality assessment were small sample size, lack of inclusion and exclusion criteria, measures not validated, and reliability tested. Considering the small number of patients with rare diseases, studies that included more than 15 patients were considered to meet the sample size requirement; however, six studies did not meet this requirement. Among clinical trials, open-label trials were the most common.

### Clinical trials

Three clinical trial studies were reviewed, including two pharmaceutical clinical trials (miglustat and eliglustat) [25, 26] and one evaluating the effects of iron chelation therapy [27]. All clinical trials used the SF-36 as a clinical outcome assessment measure, and one trial applied the Fatigue Severity Scale.

In a 24-month, randomized, open-label phase II study, 36 patients with GD-1 of varying clinical severity were enrolled in the study to examine the safety and efficacy of oral miglustat [25]. The 24-month period included a 6-month randomized trial and an 18-month extension period. A total of thirty-six patients were randomly assigned to three intervention groups (miglustat alone, imiglucerase + miglustat, and imiglucerase alone) and their QoL was assessed using the SF-36. At 6-month, there was a significant difference in the mean changes from baseline in SF-36 Mental Health between patients receiving miglustat (who improved) and those receiving imiglucerase or combination therapy (who deteriorated). Additionally, the miglustat group reported greater convenience and satisfaction with the treatment.

Another study reported the final 8-year outcomes of previously untreated 19 adults with GD-1 who completed an open-label phase II trial of eliglustat. The administered QoL measures included the SF-36 and Fatigue Severity Scores [26]. The mean QoL and disease severity improved significantly during the first 3–4 years of eliglustat treatment, with only slight changes in the values during the remainder of the study period. Among the 16 patients with baseline and 8-year values, the mean (± standard deviation [SD]) of Fatigue Severity Score (1 = least severe, 7 = most severe) had decreased by 24% from 4.44 ± 1.79 to 3.28 ± 1.62 at 8 years.

The last open-label RCT analyzed the data of eight patients with GD, and the QoL was measured by the SF-36 after the patients received two iron chelation therapies (deferasirox or deferoxamine). At 4 weeks and 4 months, there was no significant difference between the two iron chelation therapies in terms of the patients’ QoL physical component scores [27].

### Cohort studies

The present review includes two longitudinal studies, a retrospective study [28] and prospective [29]. The 12 months retrospective cohort study was conducted to investigate the differences in the clinical and subjective well-being of 34 GD-1 patients experiencing ERT dosage reduction after a forced temporary imiglucerase shortage. The results showed that drug reduction did not induce a substantial modification in the laboratory values but seemed to have influenced the perception of well-being of some GD patients [28]. The prospective cohort study included 48 patients with GD who underwent total hip replacement. QoL, hip function, and pain were assessed using the EQ-5D, hip-related disability (HHS) score, and visual analog scale (VAS), respectively. Strong linear correlations were found among the indices themselves, that is, between HHS and VAS (R = 0.505), HHS and EQ-5D (R = 0.88), and EQ-5D and VAS (R = 0.614) [29].

### Cross-sectional studies and qualitative research

We included eighteen cross-sectional studies and two qualitative studies. These studies focused on evaluating the QoL of patients with GD and analyzing the influencing factors associated with the patients’ QoL, both positive and negative.

GD severely affects several major QoL dimensions [7, 13, 19, 20, 30–35]. Patients with GD scored significantly worse than did the age- and sex-adjusted normal population on five of the eight SF-36 subscales (*p* < 0.05) [20]. The median health status score on the EQ-5D for patients with GD in the UK was 0.727 (confidence interval [CI], 0.691–0.796), with three patients having a health status score < 0 [30]. The results showed that physical symptoms, such as bone pain, chronic fatigue, bleeding, and splenomegaly, can cause patients to exhibit moderate to severe psychological complications (e.g., anxiety, depression, and feelings of isolation) that interfere with daily life, school, work, and social activities [13]. Moreover, qualitative studies have found that experience a variety of stresses, and that discomfort, inconvenience, and the high cost of treatment can also cause psychological problems for patients [6, 13]. A study found that although children’s and parents’ PedsQL 4.0 scores were consistent (i.e., the coefficients for internal consistency exceeded 0.70 for the majority of the subscales in both self-report and parent proxy-report versions), the pattern of association between symptoms and perceived burden was different for children and parents [7]. In children, the presence of symptoms such as bone, joint, or abdominal pain had a significant impact on the reported QoL; however, the QoL was more significantly affected by frequent or abnormal bleeding and fatigue in children in parent proxy reports [7].

Several cross-sectional studies have identified factors associated with the QoL of patients with GD [6, 7, 13, 20, 30, 32, 34–37]. ERT treatment is the most important factor for improving the QoL of patients, and the earlier it is received, the more significant the effect [35]. Bone, joint, or abdominal pain; bleeding; joint replacement; spleen replacement; and fatigue have a negative impact on the QoL [7, 13, 20, 36]. In clinical practice, it is necessary to distinguish between bone pain and neuropathic pain in patients with GD in order to consider the most appropriate disease management and facilitate patient care and prognosis [38]. A study in Bulgaria reported a statistical correlation between the cost of medication and QoL [31]. The high incidence of neurological symptoms in patients may be related to concurrent medical problems and/or the side effects of concurrent medications [39]. Parkinsonism and other neurological symptoms may be a significant burden for patients with GD; however, symptomatic management can improve their QoL [40]. Type 2 and 3 GD are often associated with neurological involvement and symptoms such as dysphagia, dyspnea, epilepsy, Parkinson’s disease, and cognitive decline. Cognitive impairment and depression may be early predictive factors for Parkinsonism in the GD population [36]. The vitality and neurological symptoms in patients with GD are also significantly affected, and daytime sleepiness is a common symptom [40].

### Pre- and post-intervention investigations

Seven studies evaluated changes in the QoL before and after ERT/SRT treatment [14, 15, 41–45], and one study also considered acupuncture [45]. The SF-36 was the most commonly used instrument, while the Lansky Play-Performance Scale was used to assess treatment the outcomes in children. After ERT/SRT treatment, patients with GD experienced a reduction in bleeding, chronic fatigue, gastrointestinal discomfort, and bone pain, and a significant improvement in psychosocial functioning [15, 41, 42, 44]. The SF-36 showed an improvement in vitality (energy level and fatigue) first [15]. Bone pain was relieved after treatment but remained an important influencing factor for the QoL [14, 44], and the psychological status did not improve significantly after the intervention [15]. The use of ERT every two weeks showed substantial benefits and significantly improved the QoL, assessed with the Lansky Score, in five children with GD-1 [43]. Acupuncture, an ancient Chinese therapy, has been used to treat patients with GD. A total of 12 patients participated in the treatment, and while the only pain outcome reduced by acupuncture was knee pain, significant improvements were observed in almost all FACIT fatigue measures [45].

### Validation and introduction of a disease-specific scale

GD-specific scales have been developed, including two applicational versions: a 24-item version for routine clinical monitoring (rmGD1-PROM) and a 17-item version for clinical trials (ctGD1-PROM), with psychometric properties measured using the ctGD1-PROM [21]. The instrument was developed in three countries (US, France, and Israel) and resulted in three versions in: Hebrew, Arabic, and English. The rmGD1 PROM was used in a cross-sectional survey in 2020 [46]. The use of a GD-1 specific PROM highlights personal problems that are not captured by traditional outcome parameters (i.e., GD1-related restrictions and concerns, fatigue, physical weakness, bone pain, and worry regarding the future) [46]. The psychometric results showed strong evidence of convergent validity based on correlations between the overall and item-level ctGD1-PROM scores and summary scores of the physical and mental components of the SF-36 [21]. In addition, the internal consistency of the ctGD1-PROM was excellent (Cronbach’s alpha = 0.928) [21].

## Discussion

Our review analyzed 33 studies pertaining to patients with GD, which incorporated patient-reported outcomes and were published after 1993. Notably, the number of publications per annum has demonstrated a consistent and upward trajectory, rising from a mean of 0–1 publication yearly to 3–5 publications annually in recent times. Indeed, this tendency is related to the importance given to QoL by official health technology assessment bodies, and for rare diseases, the role of QoL in clinical trials and disease management has become more prominent [47, 48]. Nonetheless, this escalation in research output has revealed heterogeneity in research methods, instruments, and conclusions. Given the absence of widely accepted QoL instruments for GD, and with only one such instrument currently in circulation, researchers have employed various QoL instruments to capture the salient constructs of interest, often with overlapping domains.

GD is a rare disease, and its singular features are evident in the present study. Notably, the scarcity of patients with GD poses a challenge to patient recruitment [48, 49]. The majority of the studies incorporated in our review recruited patients via hospital-based clinical experts or patient groups. It is worth highlighting the considerable variation in patient numbers among the included studies, ranging from 3 to 212. Additionally, the heterogeneity of GD engenders marked inter-patient dissimilarities with regard to the clinical presentation, initial manifestations, and disease progression [50]. This variability is manifested in the diverse clinical presentation among the three GD types, as well as among patients of the same type with distinct underlying conditions. These factors have contributed directly to the observed discrepancies between patient experiences and self-reported outcomes. Furthermore, the scarcity of treatments for GD and ethical infeasibility of placebo groups necessitate the implementation of single-arm or pre-post-controlled trials, prompting judicious consideration of factors such as the sample size, patient demographics, age, and geographic location, as well as the instruments employed when interpreting and applying PROs results to GD patients. In this respect, attention must be focused on the most prevalent symptoms and effects that are of primary significance to patients, and which are expected to be alleviated or stabilized following treatment.

The measurement of QoL in GD patients is dominated by generic instruments, and disease-specific scales are not sufficiently used. The GD1-PROM was developed based on patients from three countries, which largely expanded the number of patients included, ensured the quality of the cognitive interview, and avoided the omission of critical symptoms [21]. However, there may be differences between patients in different countries in terms of the genetic phenotype, language, culture, and perception of illness, which may also influence later administration of the scale. Currently only one of the two versions of the GD-PROM has been psychometrically validated; the other is applicable to routine monitoring in clinical practice and has not been validated. Finally, in some countries, a GD disease-specific scale cannot be directly applied because of language or cultural adjustment problems. In situations where the application of disease-specific scales is restricted, the use of a combination of symptom and generic scales may be a better solution.

The inclusion of PROs in treatment trials for patients with GD has the potential to provide unique and valuable information to facilitate medical decision-making [51]. However, there are potential methodological challenges in the study design and implementation that must be adequately addressed. On the one hand, previous studies examining PROs in patients with GD have been predominantly cross-sectional or pre- and post-intervention investigations, and all three clinical trials have been open-label, with limited confidence in the conclusions drawn and a failure to effectively control for confounding factors. On the other hand, some studies have included patients without differentiation of subtypes, leading to a wide variation in results. Future studies must optimize the study design by utilizing a more uniform patient population and conducting subgroup analyses according to patient age and treatment intensity to ensure the accuracy of the assessment. Furthermore, the study design for PROs must also consider parent proxy responses and self-reporting of affected children. In instances where GD patients are too young or unable to complete self-reports because of illness or cognitive impairment, parents may be asked to report their child’s QoL through a parent proxy report. Studies on patients with GD, similar to those conducted on other rare [52, 53] and non-rare diseases [54, 55], have demonstrated moderate to good agreement between child self-reports and parent proxy reports. However, it is important to note that child’s and parent’s perceptions of the aspects of the disease affecting the QoL may differ. Therefore, future comprehensive assessments should incorporate both child and parent perspectives [56].

The application of PROs in GD has primarily focused on evaluating the current QoL and effectiveness of post-treatment interventions. However, more extensive applied studies, such as patient health utility values, satisfaction with treatment, and adherence to treatment, which contribute to drug development and marketing, are lacking. PROs are now widely used in the evaluation of new drugs, with 20% of new drug labels between 2006 and 2015 including PRO endpoints [47]. The fact that most rare diseases are chronic and require long-term intervention, and that clinical endpoints are not well-defined, highlights the value of PROs in rare diseases [48, 49]. In recent years, some countries and regions, such as the European Union and United States, have made increasing efforts to incorporate the patient voice into drug development. Between 2012 and 2016, orphan drug approvals by the European Medicines Agency and U.S. Food and Drug Administration were mainly focused on rare drugs, with 21.7% and 9.0% of all approved orphan drugs applying for PROs, respectively [57, 58]. Therefore, the use of PROs should be expanded to include studies of reported outcomes in patients with GD in order to better help patients in making decisions about disease management and health technology assessment.

## Conclusion

The use of PROs in GD has been receiving increasing focus and attention, as evidenced by the upward trend in the number of studies conducted since 2016. Although a few disease-specific scales have been developed and validated, generic instruments such as the SF-36 are still primarily used for PROs assessment in GD. However, the measurement of QoL in GD is complicated by factors such as the small number of patients, disease heterogeneity, and cross-regional studies. To improve the measurement of patient QoL and treatment effectiveness, reliable and valid PROs instruments that reflect the unique experiences of patients with GD are needed. Ultimately, the incorporation of PROs in GD research and clinical practice can provide valuable insights for patients and healthcare professionals, supporting informed decision-making and improving patient outcomes.

### Electronic supplementary material

Below is the link to the electronic supplementary material.

**Additional file 1.** Search terms.

## Data Availability

Not applicable.

## Abbreviations

GD: Gaucher’s disease
QoL: Quality of life
SRT: Substrate-reduction therapy
ERT: Enzyme replacement therapy
PROs: Patient-Reported Outcomes
PROMs: Patient-reported outcome measures
RCT: Randomized controlled trial
PRISMA: Preferred reporting items for systematic reviews and meta-analyses
