# Supplementary material for: Patient-reported outcomes in Gaucher’s disease: a systematic review
Source: Orphanet J Rare Dis. 2023 Aug 25;18:244. doi: 10.1186/s13023-023-02844-w (PMC10463869; doi:10.1186/s13023-023-02844-w)
Supplement: Supplementary file 1 — Additional file 1. Search terms. [file 13023_2023_2844_MOESM1_ESM.docx]

**Search strategy**

1. **Search terms**

| **Patient reported outcomes related terms** | **GD related terms** |
| --- | --- |
| Patient reported outcomes / PRO / PROs | Gaucher Disease |
| Health related quality of life **/** HRQoL | Gaucher's Disease |
| Quality of life **/** QoL | Gauchers disease |
| Outcomes Assessment | Gaucher |
| quality-adjusted life years | GD |
| Quality of life or health related quality of life assessment |  |
| Quality of life or health related quality of life survey |  |
| Cognition / cognitive / fatigue / pain |  |

1. **Search strategy**

‘Patient reported outcomes’ related AND ‘GD related terms’

1. **Examples of Pubmed search**

| **Database** | **Search Strategy** |
| --- | --- |
| **PubMed** | #1 Search: "Gaucher Disease"[Mesh] Sort by: Most Recent  #2 "Gaucher's Disease"[Title/Abstract] OR "Gaucher Disease"[Title/Abstract] OR "Gauchers disease"[Title/Abstract] OR "Gaucher"[Title/Abstract]  **#3 #1OR#2**  #4 "Patient Reported Outcome Measures"[MeSH Terms]  #5 "Quality of Life"[MeSH Terms] OR "Quality-Adjusted Life Years"[MeSH Terms]  #6 "PRO"[Title/Abstract] OR "PROs"[Title/Abstract] OR "patient-reported outcomes"[Title/Abstract] OR "patient report outcome"[Title/Abstract] OR "Outcome Assessment"[Title/Abstract] OR "qaly"[Title/Abstract] OR "qualy"[Title/Abstract] OR "quality adjusted life"[Title/Abstract] OR "quality adjusted life year"[Title/Abstract] OR "quality-adjusted life years"[Title/Abstract] OR "quality of life"[Title/Abstract] OR "QOL"[Title/Abstract] OR "Life Quality"[Title/Abstract] OR "health related quality of life"[Title/Abstract] OR "health related quality of life"[Title/Abstract] OR "hrqol"[Title/Abstract] OR "hrqol"[Title/Abstract] OR "health status"[Title/Abstract]  #7 "well-being"[Title/Abstract] OR "well-being"[Title/Abstract] OR "anxiety"[Title/Abstract] OR "depression"[Title/Abstract] OR "pain"[Title/Abstract] OR "function"[Title/Abstract] OR "activity"[Title/Abstract] OR "joint status"[Title/Abstract] OR "symptom"[Title/Abstract] OR "cognition"[Title/Abstract] OR "cognitive"[Title/Abstract] OR "sleep"[Title/Abstract] OR "burden"[Title/Abstract] OR "fatigue"[Title/Abstract] OR "psychology"[Title/Abstract] OR "patient satisfaction"[Title/Abstract]  **#8 #4OR#5OR#6OR#7**  **#9 #3 AND#8** |
